# Results of the 2019 Survey of Engineered Nanomaterial Occupational Health and Safety Practices

**DOI:** 10.3390/ijerph19137676

**Published:** 2022-06-23

**Authors:** Nicole M. Neu-Baker, Adrienne Eastlake, Laura Hodson

**Affiliations:** 1 College of Nanoscale Science & Engineering, Nanobioscience Constellation, State University of New York (SUNY) Polytechnic Institute, Albany, NY 12203, USA; neubakn@sunypoly.edu; 2 National Institute for Occupational Safety and Health (NIOSH), Cincinnati, OH 45226, USA; lhodson@cdc.gov

**Keywords:** nanotechnology, occupational health, worker safety, exposure, risk factors, questionnaires, statistical analysis, environmental monitoring, engineering controls, training, sampling, personal protective equipment

## Abstract

In collaboration with RTI International, the U.S. National Institute for Occupational Safety and Health (NIOSH) administered a survey to North American companies working with nanomaterials to assess health and safety practices. The results would contribute to understanding the impact of the efforts made by the NIOSH Nanotechnology Research Center (NTRC) in communicating occupational health and safety (OHS) considerations for workers when handling these materials. The survey, developed by RAND Corporation, was conducted online from September 2019–December 2019. Forty-five companies or organizations in the U.S. and Canada that fabricate, manufacture, handle, dispose, or otherwise use nanomaterials completed the survey. The survey was designed to answer research questions regarding the nanomaterials in use, which resources the companies have consulted for OHS guidance, and the overall OHS culture at the companies. Other questions specifically addressed whether the companies interacted with NIOSH or NIOSH resources to inform OHS policies and practices. Among participating companies, 57.8% had a maximum of 50 employees. Gold nanoparticles and polymers were most common (n = 20; 45.5% each), followed by graphene (36.4%), carbon nanotubes and nanofibers (34.1%), and zinc oxide nanoparticles (31.8%). Environmental monitoring was performed by 31.8% of the companies. While 88.9% of the companies had laminar flow cabinets, only 67.5% required it to be used with ENMs. Information and training programs were indicated by 90% of the sample, and only 29.6% performed specific health surveillance for ENM workers. Personal protective equipment primarily included gloves (100%) and eye/face protection (97.7%). More than a third (37.8%) of the respondents reported using at least one NIOSH resource to acquire information about safe handling of ENMs. The small number of companies that responded to and completed the survey is a considerable limitation to this study. However, the survey data are valuable for gauging the reach and influence of the NIOSH NTRC on nano OHS and for informing future outreach, particularly to small businesses.

## 1. Introduction

The U.S. National Institute for Occupational Safety and Health (NIOSH) established the Nanotechnology Research Center (NTRC) in 2004 to identify critical issues related to potential worker exposure to nanomaterials. The NTRC is charged with creating a strategic plan for investigating these issues, coordinating the NIOSH research effort, establishing research partnerships, and disseminating information. For over 15 years, NIOSH has made an investment in research promoting the responsible development and application of engineered nanomaterials (ENMs). As a result, NIOSH has issued occupational health and safety (OHS) guidance documents and informational resources [1,2,3,4,5,6,7,8,9,10,11,12,13,14,15] for industries and workers that use or handle nanomaterials. Other U.S. government agencies, including the Environmental Protection Agency (EPA), Occupational Safety and Health Administration (OSHA), and National Institute of Standards and Technology (NIST), have also published OHS guidance documents [16,17,18,19], as have non-governmental entities and organizations, such as the World Health Organization (WHO), industry associations, and product manufacturers [20,21]. NIOSH also offers on site health and safety evaluations for companies using ENMs. This service is provided free of charge to the volunteer companies that elect to coordinate a site visit and includes written recommendations specific to the company. However, the extent to which companies follow these written recommendations is unknown [22,23].

NIOSH is engaged with the nanotechnology community in other ways, via conference attendance, invited talks, and teaching professional development courses. These avenues allow for more interpersonal communication of OHS concerns and can immediately and directly disseminate information and guidance to the community at large. These interactions also provide real-time feedback to NIOSH on the most pressing needs, issues, or questions surrounding nano OHS. Formal research partnerships between NIOSH and universities, industry, and other government agencies, contribute to these efforts.

In an effort to assess the impact of the NIOSH guidance, NIOSH sponsored a survey of engineered nanomaterial OHS practices. The survey questionnaire was prepared under contract with RAND Corporation and administered in collaboration with RTI International from September 2019–December 2019 with companies and organizations in the U.S. and Canada working with ENMs. The survey was designed to answer research questions including, but not limited to the types and forms of ENMs in use; the sources of information companies use for health and safety guidance; how companies handle ENMs; the OHS practices in use; and the companies’ level of organizational commitment to ENM OHS. Survey findings were used to inform NIOSH and guide the NTRC in future OHS outreach to companies that handle ENMs. A previous survey by Iavicoli et al., 2019 looked at risk management practices in the EU and this survey compliments those results by looking at US and Canadian companies.

## 2. Materials and Methods

### 2.1. Survey of Engineered Nanomaterial OHS Guidance

#### 2.1.1. Identification of Companies

RTI and NIOSH identified 601 companies and other organizations, including universities and government agencies (referred to collectively as “companies”), that met study eligibility criteria. The target population consisted of companies and other organizations in the U.S. or Canada that manufacture, distribute, fabricate, formulate, use, or provide services related to ENMs. The list of potential companies was developed from existing lists created by industry associations, marketing databases, researchers, and from targeted web searches. These included the Consumer Products Inventory, National Science Foundation (NSF) and National Institutes of Health (NIH) grantees (to identify universities receiving funding for ENM research), Nanowerk’s Nanotechnology Company and Research Labs Directory, UnderstandingNano directory, NanotechnologyNow directory, Silver Nanotechnology Commercial Inventory, Alibaba.com, and chemeurope.com. At each company, one person was contacted and asked to identify the best person who was knowledgeable about the safety and health programs at the company to participate in the survey.

#### 2.1.2. Questionnaire and Survey Procedures

The survey was launched on 19 September 2019, and the data collection period ended on 31 December 2019. NIOSH’s 35-item questionnaire was designed to capture information to address ENM OHS practices and understanding the influence of NIOSH’s efforts on nano OHS (see Supplementary Materials). RTI programmed the survey instrument using Voxco Online software installed on secure servers to ensure secure data collection. RTI programmers created a case management system to store, access, and manage the sampled companies and their contact information. This system managed calls, email invitations, and reminders, and tracked survey completion status. Each company was categorized into one of three tailored scenarios based on the contact information that was available: phone, email, or LinkedIn. For 76 cases, trained interviewers made phone calls to companies to determine eligibility and identify a point of contact. Eligible cases moved into the email condition. RTI sent an introductory email to 420 companies, followed by an email invitation and two reminder emails (sent in two-week intervals) through the case management system. For some commercial companies without reliable phone or email contact information, LinkedIn was used as a potential method of initial contact. RTI professional staff sent tailored messages through LinkedIn to 51 companies with each company’s tailored link to the web survey. In addition to the direct data collection efforts, NIOSH promoted the survey through outreach activities, including in the September 2019 edition of NIOSH eNews and an October 2019 science blog post.

## 3. Results

### 3.1. Eligibility and Response Rates

**Figure 1:**
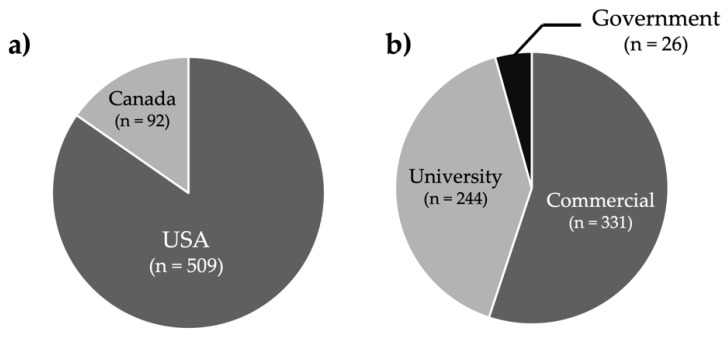
Location and Sector of Identified Companies. (**a**) Of the 601 companies identified, 509 were located in the U.S. and 92 were located in Canada. (**b**) Of the 601 companies identified, 331 were in the commercial sector, 244 were universities, and 26 were governmental organizations.

The 601 companies identified were then classified by sector: commercial, university, or government (Figure 1). Of the 601 companies identified and contacted for the survey, 28 were ineligible because they were either no longer in business or did not work with ENMs. Out of the 573 remaining companies, 57 responded to the survey, for a response rate of 9.5%. Eligibility could not be confirmed for the 431 companies that did not respond to phone calls or emails. Of the 57 companies that responded, 45 companies completed the survey.

### 3.2. Participating Companies

The percentages presented in the tables are based on 45 total respondents, unless otherwise noted. Most participating companies (n = 37; 82.2%) had fewer than 250 people working at the locations sampled, and over half of the participating companies (n = 26; 57.8%) employed 50 or fewer individuals at their location(s). Only four participating companies (8.9%) had more than 1000 employees at the sampled location.

**Figure 2:**
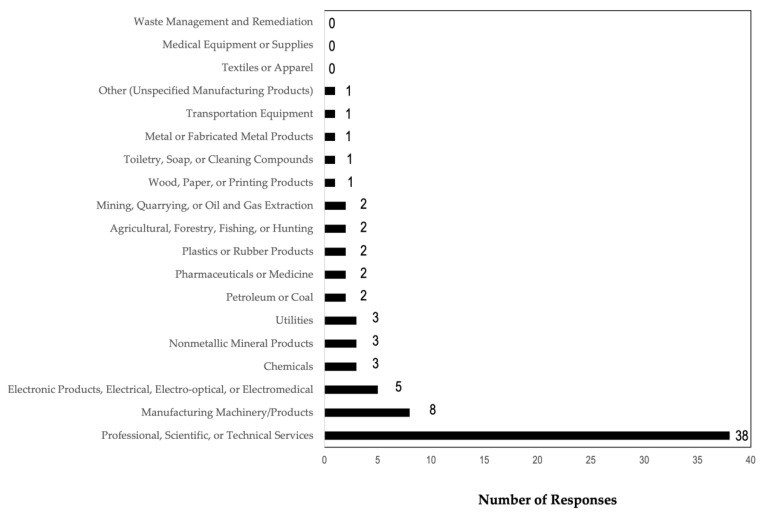
Commercial Sector for ENM Use. The majority of the companies reported ENM use for professional, scientific, or technical services (n = 38; 84.4%). Note: multiple responses allowed; missing n = 1.

Over 80% (n = 38; 84.4%) of the companies who completed the survey were working with ENMs intended to be used in the professional, scientific, or technical services sector (Figure 2). Most of the companies (34 of 45) had three or fewer employees or contractors that had any contact with ENMs. No participating company had more than six individuals in contact with ENMs. Most participating companies (n = 40; 88.9%) handled less than a kilogram of ENM on a daily basis, and on average, only a few employees at a given location had any contact with ENMs.

### 3.3. ENMs Handled by Participating Companies

The respondents were asked about the physical forms and different types of ENMs the companies handled. Participating companies handled ENMs in each of the physical forms: suspended in liquid (n = 36; 80.0%) followed by dry powders (n = 31; 68.9%). More than half reported handling ENMs as solids (n = 25; 55.6%) and suspended in a matrix (n = 24; 53.3%). Only four companies (8.9%) worked with ENMs contained in an aerosol. The percentages indicate that companies worked with ENMs in more than one physical form (multiple responses to this question were allowed).

Nearly two-thirds (n = 29; 65.9%) of the companies worked with nanoparticles (as opposed to nanofibers, nanosheets, dendrimers, etc.) (Supplemental Table S1). Gold nanoparticles were most common (n = 20; 45.5%), followed by zinc oxide nanoparticles (31.8%), titanium dioxide nanoparticles (29.5%), and silver nanoparticles (25.0%). More than half of the companies worked with nanotubes, nanofibers, nanorods, or nanowires (52.3%). About a third of the companies worked with multi-walled carbon nanotubes (34.1%) and single-walled carbon nanotubes (31.8%). Over a third of the companies worked with polymers (45.5%) or nanosheets (36.4%). Most companies who worked with nanosheets worked with graphene (36.4% of all companies). Less commonly used materials included nanocrystalline cellulose (13.6%), nanofibrils cellulose (9.1%), dendrimers (9.1%), and nanoclays (4.6%). Many companies reported working with more than one type of ENM (multiple responses to this question were allowed).

Most companies developed ENMs (n = 36; 80.0%) or used ENMs for developing products or applications (n = 28; 62.2%). Other companies used ENMs for manufacturing (n = 18; 40.0%), incorporating ENMs into their products (n = 17; 37.8%), and conducting laboratory scale-up of ENMs (n = 15; 33.3%). The least common uses reported by the companies were for providing services using products containing ENMs (n = 8; 17.8%); repackaging and distribution of ENMs (n = 6; 13.3%); and for production of instruments for the manufacturing, characterization, and detection of ENMs (n = 2; 4.4%). Two companies (4.4%) reported “other ENM research”. Most companies completed their own material characterization (n = 39; 86.7%). None of the companies reported using ENMs for waste management or remediation, medical equipment or supplies, or for textiles or apparel. Note that this question allowed for multiple responses.

### 3.4. Occupational Health and Safety (OHS) Culture at the Responding Companies

#### 3.4.1. Organizational OHS Expertise

Most participating companies (n = 37; 84.1%) had an OHS office, department, or individual working in an OHS capacity. Respondents reported the total number of years they had personally worked with ENMs. The median was 10 years, and the highest number reported was 40 years. More than three-quarters of the respondents (n = 34; 75.6%) had more than five years of experience. The respondents held varied roles in their companies and most (n = 26; 66.4%) reported holding leadership positions. Nearly a third (n = 13; 30.2%) were executive leaders and almost another third (n = 13; 30.2%) were directors or managers. A quarter of respondents (n = 11; 24.4%) were in academia. The remaining respondents were safety and regulatory officers (n = 6; 14.0%). Most respondents had at least some level of OHS responsibility (n = 39; 86.7%). Those with certifications in OHS (n = 9; 20.9%) reported being Certified Industrial Hygienists (CIHs), Certified Safety Professionals (CSPs), or Certified Hazardous Materials Managers (CHMM), and had completed OSHA 30-Hour training and Workplace Hazardous Materials Information System training

#### 3.4.2. Employee OHS Training

Three-quarters of respondents (n = 33; 75.0%) reported that the workforce at their companies received training on the safe use or handling of ENM. The training was supplied by external training resources or consultants (n = 32; 97.0%), internal informal training (n = 22; 66.7%), and internal, formal training (n = 6, 18.2%). Note that the question on training allowed for multiple responses.

**Figure 3:**
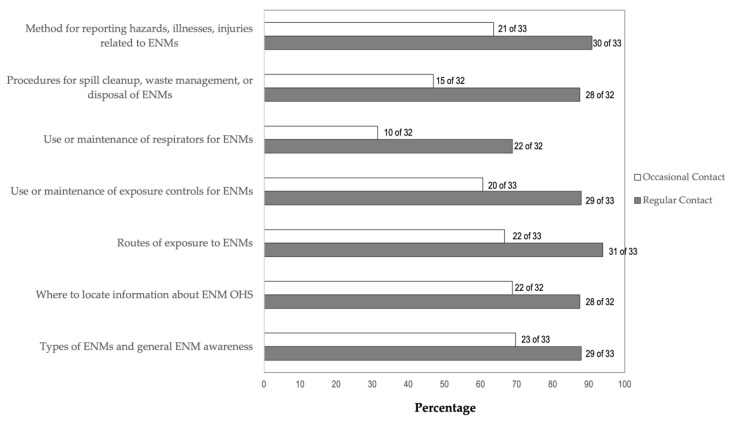
OHS Training for Employees with Regular or Occasional Contact with ENMs. OHS training topics for employees with regular contact (gray bar) or occasional contact (white bar) with ENMs. For all training topics listed, a higher percentage of employees with regular contact with ENMs received training compared to those with occasional contact.

At companies that provided training, the level of an employee’s contact with ENMs was related to their likelihood of receiving training on the safe use or handling of ENMs. For each type of training the survey asked about, a higher percentages of staff who had regular contact with ENM received the training compared to staff with only occasional contact with ENMs. Staff who had no contact with ENMs were less likely than staff with some contact to receive training on their safe use and handling. For example, 93.9% of companies (31 of 33) provided training on the routes of exposure to ENMs to employees who had regular contact with ENM compared to 66.7% of companies (22 of 33) who provided this training to employees with occasional contact with ENM (Figure 3). Only 18.2% of companies provided training on routes of exposure to ENM to employees who had no known or intended contact with ENMs.

Nearly 90% of companies provided training on each of the other topics asked about for employees who had regular contact with ENMs: methods for reporting hazards, illnesses, and injuries related to ENMs (90.9%); types of ENMs and general ENM awareness (87.9%); use or maintenance of exposure controls (87.9%); location of ENM safety and health practices information (87.5%); ENM spill cleanup, waste management, or disposal procedures (87.5%). The exception was training on the use or maintenance of respirators, for which 68.8% of companies provided to employees who had regular contact with ENMs. This percentage could be considered high because only 36.4% (n = 16) of companies reported that employees who work with ENM use respirators.

To get a more complete picture of participating companies’ commitment to ENM safety, the survey included questions about the extent to which companies assessed employees’ knowledge of ENM safety and monitoring of potential exposures to ENM. Only 34.1% (n = 15) of participating companies measured their employees’ knowledge of ENMs. Among companies that did assess employee ENM awareness and knowledge, some used regular inspections (n = 8; 53.3%) or periodic surveys (n = 7; 46.7%), and some used a combination of strategies (n = 6; 40.0%).

#### 3.4.3. OHS Practices and Guidelines Followed

All respondents reported that their companies implemented a safety and health program for employees. All the respondents also reported that their companies use exposure controls (elimination, substitution, engineering, administrative, personal protective equipment). Respondents indicated whether 15 types of health and safety practices, not specific to ENMs, were used at the companies. Over half of the respondents indicated that 13 of the 15 practices were used at their companies (Supplemental Table S2). Only 31.8% (n = 14) did exposure monitoring and 29.6% (n = 13) conducted medical screening and surveillance.

In the absence of specific ENM OHS guidelines, responding companies reported that they used guidance for general chemical hazards (n = 21; 47.7%) or biohazards (n = 12; 27.3%) to inform their safety and health practices.

#### 3.4.4. Engineering Controls

The survey asked about the use of 12 different types or categories of engineering controls commonly employed for chemical protection. The largest percentages of companies used laminar low-flow ventilated enclosures (n = 40 of 43 respondents; 88.9%), separate work areas like control rooms (n = 29 of 40 respondents; 70.7%), high-efficiency particulate air (HEPA) filtration (n = 26 of 40 respondents; 65.0%), and handling ENMs in a slurry or suspension as opposed to handling ENMs as dry powders (n = 26 of 42 respondents; 61.9%) (Supplemental Table S3).

#### 3.4.5. Personal Protective Equipment (PPE)

In addition to the engineering controls, the employees who work with ENMs at all participating companies used gloves (Supplemental Table S4). Nearly all the respondents also used eye or face protection (n = 43; 97.7%) and coveralls or lab coats (n = 40; 90.9%). About a third of companies (n = 16; 36.4%) reported using respirators. More than a quarter of companies used shoe covers (n = 13; 29.6%) and hair bonnets (n = 12; 27.3%).

#### 3.4.6. Changes Made to Existing OHS Practices to Accommodate Working with ENMs

As mentioned in Section 3.4.3, the questionnaire asked respondents to first indicate the specific health and safety practices used at their companies (Supplemental Table S2) and for each practice they selected, the survey asked if they have separate or specific guidance for ENMs (Table 1). The percentages shown in Table 1 are based on the number of respondents who answered each item after they first indicated that their company used the more general form of the safety practice. Over a third of companies had separate or specific guidance for ENM for each of the more general health and safety practices they had in place (Table 1). The largest percentage of companies with specific guidance for ENMs included determining routes of exposure (n = 23 of 38; 60.5%), followed by identifying processes or tasks where workers might be exposed, and evaluating new processes and procedures for hazards. Fewer companies had specific guidance for ENMs for spill cleanup, medical screening and surveillance, exposure monitoring and reporting hazards, illness, or injuries.

As mentioned in Section 3.4.4, the questionnaire asked respondents to first indicate the engineering controls used at their companies for any chemical or material hazard (Supplemental Table S3), and for each control selected, the respondents were asked if it was required when working with ENMs (Table 2). The percentages shown are based on the number of respondents who answered each item after they first indicated that their company used the more general form of the engineering control. For example, 67.5% (n = 27) of the 40 companies who reported that they had a laminar low-flow enclosure required this engineering control for working with ENMs. Most commonly, companies required use of separate work areas, ultra-low particulate air (UPLA) filtration or HEPA filtration, separate HVAC system, or working with nanomaterial in a slurry or suspension instead of dry powder.

#### 3.4.7. Process Emission or Exposure Monitoring

**Figure 4:**
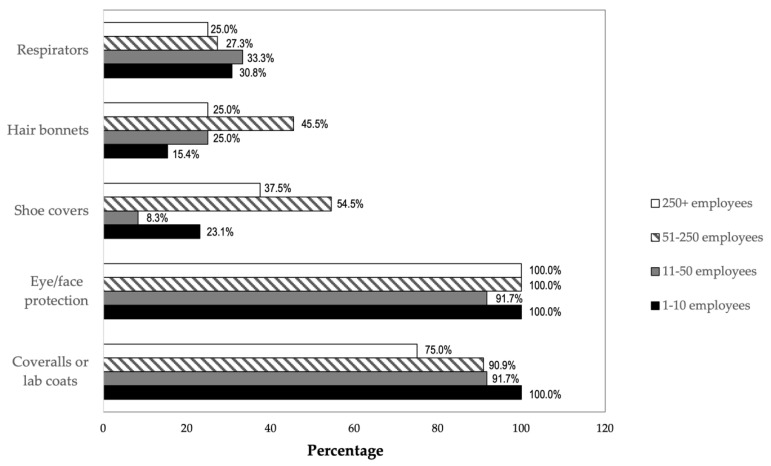
Process Emission or Exposure Monitoring. Note: multiple responses allowed; missing n = 1.

Most of the participating companies did not do any process emission or exposure monitoring (n = 28; 63.6%; Figure 4). However, companies that did monitor exposure tended to use direct reading particle counters, filter-based sampling, and/or wipe sampling (multiple answers were allowed). Among the 16 companies that conducted exposure monitoring, most reported doing it periodically (n = 12; 75%), although some companies monitored more than one of the timing options, such as with changes in processes or controls or in response to a spill or an unanticipated event.

### 3.5. OHS Resources Used

#### 3.5.1. NIOSH-Specific OHS Resources Used

More than a third (n = 17; 37.8%) of the respondents reported using at least one NIOSH resource to acquire information about safe handling of ENMs (Supplemental Table S5). The most frequently used resource was “Approaches to Safe Nanotechnology” [2], which 36.4% (n = 16) of respondents reported using, followed by 34.1% (n = 15) who reported using “General Safe Practices for Working with Engineered Nanomaterials in Research Laboratories” [5]. The more specific NIOSH publications, “Occupational Exposure to Carbon Nanotubes and Nanofibers” [13] and “Occupational Exposure to Titanium Dioxide” [12], were used by 20.5% (n = 9) and 18.2% (n = 8) of respondents, respectively. Three respondents (6.8%) reported attending a course or webinar taught by NIOSH instructors. Among the 17 respondents who used NIOSH information resources, 16 reported using more than one. On average, these 17 respondents reported that they used 3.4 NIOSH resources. A larger percentages of respondents reported that they had used NIOSH materials than the percentages of respondents that reported that they had used OSHA, EPA, NIST, or other government materials.

**Figure 5:**
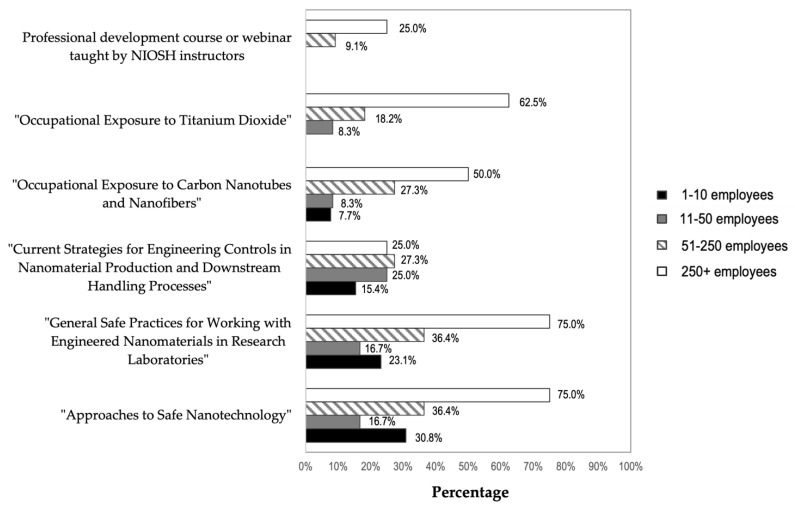
NIOSH Resources Used by Company Size. Note: multiple responses allowed. Percentages in each column are based on the number of respondents in each category: 1–10 employees = 13 respondents; 11–50 employees = 12 respondents; 51–250 employees = 11 respondents; 250+ employees = 8 respondents.

Figure 5 displays the percentage of companies within each size category that reported using the different types of NIOSH information resources listed in the survey and in Supplemental Table S6. The percentages are based on the total number of companies within each size category. For example, 75% of the eight companies with more than 250 employees reported using NIOSH “General Safe Practices for Working with Engineered Nanomaterials in Research Laboratories” [5], compared to 23.1% of the 13 companies with one to 10 employees, 16.7% of the 12 companies with 11 to 50 employees, and 36.4% of the 11 companies with 51 to 250 employees. A higher percentage of the largest companies reported using five of the six NIOSH resources, compared to the percentages of smaller companies that reported using them. None of the participating companies with 50 or fewer employees reported attending courses or webinars taught by NIOSH instructors. None of the smallest participating employers reported using the resource “Occupational Exposure to Titanium Dioxide” [12], while nearly two-thirds (62.5%) of the largest employers did.

#### 3.5.2. Other OHS Resources Used

Respondents most frequently turned to resources that were readily accessible when they needed more information about safe handling of ENMs (Supplemental Table S5) including: product manufacturer information (n = 32; 72.7%), informal discussions with professional contacts or peers (n = 25; 56.8%), government publications (n = 20; 45.5%), and web searches (n = 19; 43.2%). About a third of respondents consulted scientific and industry publications (n = 15; 34.1%) and just under a third of respondents solicited guidance at conferences and meetings (n = 13; 29.6%).

Respondents who reported receiving recommendations for handling ENM from any source were asked how they used the information. The largest percentage of respondents said the resources informed safety and health practices (n = 38; 86.4%). More than half incorporated this information into training materials (n = 27; 61.4%) and used it to inform their policies for handling ENMs (n = 26; 59.1%). Over a quarter reported using this information to modify processes (n = 15; 34.1%) or incorporate into information provided about the product (n = 12; 27.3%).

### 3.6. Site OHS Evaluations

The survey asked whether companies had hosted a site visit to evaluate and provide recommendations regarding OHS practices at any of their company locations. Among the respondents, just over a quarter (n = 12; 27.3%) had this type of site visit or consultation since 2005, although another third (n = 14; 31.8%) were not sure whether a site visit had occurred. Among the 12 who had a site visit, over half indicated they were conducted by a government organization (n = 7; 58.3%) and the remaining companies indicated they were conducted by a private company or consultant (n = 6; 50.0%). Respondents could select multiple answers and may have hosted more than one site visit. Two respondents, one in the U.S. and the other in Canada, reported that NIOSH conducted a site visit at their company. Half of the respondents who had a site visit (n = 6; 50.0%) reported that it included feedback or recommendations about handling ENMs. Most respondents (n = 5; 83.3%) who got ENM-related recommendations reported that they implemented all of the recommendations. Two-thirds (n = 8; 66.7%) of the 12 site visits were reported by small (1–10 employees; n = 4 site visits) and medium-sized (51–250 employees; n = 4 site visits) companies.

## 4. Discussion

For over a decade, NIOSH has researched and promoted responsible development and OHS practices for ENMs by publishing guidance documents [1,2,3,4,5,6,7,8,9,10,11,12,13,14,15], exposure assessment methods [24,25,26], and conducting site evaluations [27,28,29,30,31,32,33]. NIOSH has also established partnerships and collaborations with other government agencies, universities, professional organizations, and industry to further develop and disseminate OHS practices and guidelines [34]. A web-based survey was conducted to assess the impact of NIOSH’s efforts on OHS efforts by industries and organizations that utilize ENMs. Forty-five company representatives completed the survey between September 2019 and December 2019.

The participating companies reported using or handling ENMs across 15 different commercial sectors, with the large majority intended to be used in the professional, scientific, or technical services sectors. Most companies were small, with fewer than 50 employees, and none had more than six employees who work with ENMs at the locations they were reporting on. Most of the companies worked with small quantities of ENMs, typically less than 1 kg. This finding could be a function of the types of organizations that were willing to participate in a survey sponsored by NIOSH.

Most of the individuals who completed the survey reported having more than five years of experience working with ENMs and knowledge of their company’s OHS practices, which suggests that the results are based on input from experienced personnel. Respondents held varied roles in their organizations and most reported holding leadership positions. Nearly a third were executive leaders and almost another third were directors or managers. Less than a quarter of all respondents (n = 9; 20.9%) held any OHS certification, which could create a bias in the results. All respondents reported that their companies implemented a safety and health program for employees. The level of an employee’s contact with ENMs was related to their likelihood of receiving training on the safe use or handling of ENMs. For each type of training the survey asked about, higher percentages of staff who had regular contact with ENM received the training, compared to staff with only occasional contact with ENMs. Only 18% (n = 8) of companies reported providing ENM safety training to employees who have no contact with ENMs. It is important to ensure that non-technical staff, including office workers and janitorial staff, are informed of the potential hazards of ENM exposure when performing their jobs, as they may come into contact with these materials during cleaning tasks, or due to poor hygiene practices, among other potential scenarios.

All of the respondents obtained information about handling ENMs from at least one source, even if it was only product manufacturer information or informal discussions with peers. A larger percentage of the end users in the survey used NIOSH materials compared to the percentage that used other government agencies’ publications and materials. Over 80% of the companies that had used at least one NIOSH publication reported that they used information from all sources to inform both safety practices and policies for handling ENMs, which suggests the information they found was helpful. More than half of the respondents reported that they used the information from these different sources to inform their safety and health practices by incorporating it into their training materials or informing policies for ENM handling.

The survey was able to determine if NIOSH publications and resources reach small businesses. While 75% of the large companies used NIOSH guidance documents, less than 30% of the smaller companies were using the NIOSH guidance. The most frequently used NIOSH resource by all participants was “Approaches to Safe Nanotechnology” [2], followed by “General Safe Practices for Working with Engineered Nanomaterials in Research Laboratories” [5]. A larger percentage of end users from the largest companies had accessed most of the NIOSH materials listed in the survey, compared to the percentages of end users from smaller companies. None of the participating companies with 50 or fewer employees reported attending courses or webinars taught by NIOSH instructors. This may indicate that a more targeted outreach to smaller businesses may be warranted to ensure the NIOSH OHS guidance reaches them.

### Limitations

The original intent was to develop a larger inventory of companies and to select a stratified sample of 600 from the larger group to participate in the survey. However, only 573 companies met the eligibility criteria. The low response rate of less than 8% (45/573) and small number of companies represented are a limitation to interpreting the results beyond the participating companies; however, these numbers are consistent with other surveys of organizations that handle ENMs, including Iavicoli et al. [35], who recently published findings based on surveys completed by 34 primarily European and Asian companies. Further, the low response rate and small number of participating companies may introduce bias into the sample, as companies with more focus on nano OHS practices may have been more likely to complete the survey.

The small sample size hindered doing statistical analyses that might have more directly answered some of the research questions, giving a better understanding of the impact of NIOSH guidance. Obtaining better response rates from organizations that work with ENMs is necessary; however, even survey efforts with more follow-up reminders and a longer data collection timeframe have failed to achieve even modest rates (e.g., ref. [35]). Despite low response rates, there are many similarities in responses between this survey and the survey conducted by Iavicoli et al. [35] in terms of the types of participating industries, types of materials used, and types of controls employed. While both studies have limitations, they appear to either be representative of industry activity or suffer from similar selection bias. We speculate that a shorter survey, no longer than 10 min, might have made more companies willing to participate. Additionally, companies might have been more willing to participate if they perceived a more direct benefit, such as exclusive access to an information resource or repository that could be of value to them.

## 5. Conclusions

The results of the 2019 survey of engineered nanomaterial occupational health and safety practices developed by RAND Corporation and administered online by RTI International from September 2019 to December 2019 provided NIOSH with a starting point for understanding the impact of its efforts on communicating and promoting OHS practices and considerations to companies who use ENMs. Among participating companies, 57.8% had a maximum of 50 employees. Gold nanoparticles and polymers were most common (n = 20; 45.5% each), followed by graphene (36.4%), carbon nanotubes and nanofibers (34.1%), and zinc oxide nanoparticles (31.8%). Environmental monitoring was performed by 31.8% of the companies. While 88.9% of the companies had laminar flow cabinets, only 67.5% required it to be used with ENMs. Information and training programs were indicated by 90% of the sample; only 29.6% performed specific health surveillance for ENM workers. Personal protective equipment primarily included gloves (100%) and eye/face protection (97.7%). More than a third (n = 17; 37.8%) of the respondents reported using at least one NIOSH resource to acquire information about safe handling of ENMs.

The small sample size is a significant limitation of this study, preventing many direct conclusions from being made. Based on the results, future surveys may be designed to specifically target small businesses, may be shorter in length to encourage higher response rates; may focus on the reach and impact of NIOSH guidance publications, and/or may ask more nuanced questions to broaden our understanding of the impact of the NIOSH NTRC. For example, a more nuanced question might ask if organizations with certified OHS professionals treat ENMs differently than those without certifications. Future surveys could offer an incentive (such as a chance to win a gift card) and could be promoted through stakeholders, from academia to industry, non-governmental organizations and regulators, workers’ representatives, and occupational health and safety professionals. Even with a small completion rate of less than 8% (45/573), this survey provided valuable information that future NIOSH outreach needs to focus on small businesses that use or handle ENMs.

## Figures and Tables

**Table 1:** ENM-Specific Safety Practices and Guidance Used.

ENM-Specific Health and Safety Practices Used	n *	%
Determination of routes of exposure	23 (of 38)	60.5
Identification of processes or job tasks where workers may be exposed	21 (of 40)	52.5
Evaluation of new processes/procedures for hazards	19 (of 37)	51.4
Waste management/disposal procedures	20 (of 41)	48.8
Use of exposure controls (elimination, substitution, engineering, administrative, PPE)	21 (of 44)	47.7
Assessment of need for PPE	18 (of 39)	46.2
Maintenance of engineering controls	17 (of 37)	46.0
Review of purchase orders for possible hazardous materials	14 (of 31)	45.2
Assessment of effectiveness of exposure controls	12 (of 28)	42.9
Systematic review and update of safe use procedures	15 (of 37)	40.5
Development of internal company/organization exposure guidelines	9 (of 23)	39.1
Spill cleanup procedures	15 (of 39)	38.5
Medical screening and surveillance	5 (of 13)	38.5
Exposure monitoring	5 (of 14)	35.7
Method for reporting hazards, illnesses, and injuries	14 (of 40)	35.7

* Total number of respondents who answered each item shown in parentheses. The number of respondents varies because respondents were not asked the question about specific ENM guidance if they previously reported that their company did not have the more general health and safety practice in place.

**Table 2:** ENM-Specific Engineering Controls Used.

ENM-Specific Engineering Controls Used	n *	%
Laminar low-flow ventilated enclosure	27 (of 40)	67.5
Designed or separate work areas (e.g., control room)	19 (of 29)	65.5
Ultra-low particulate air (ULPA) filtration	16 (of 27)	59.3
Separate HVAC system	15 (of 26)	57.7
Pressure differentials	15 (of 27)	55.6
Working with nanomaterial in a slurry or suspension	14 (of 27)	51.9
High-efficiency particulate air (HEPA) filtration	14 (of 28)	50.0
Laboratory fume hood	11 (of 23)	47.8
Biosafety cabinet (BSC)	10 (of 21)	47.6
Cleanroom	9 (of 20)	45.0
Local exhaust ventilation (other than fume hood, BSC, or glovebox)	12 (of 27)	44.4
Glove box	6 (of 22)	27.3

* Total number of respondents who answered each item shown in parentheses. The number of respondents varies because respondents were not asked the question about specific ENM guidance if they previously reported their company did not have the more general health and safety practice in place.

## Supplementary Materials

The following supporting information can be downloaded at: https://www.mdpi.com/article/10.3390/ijerph19137676/s1, Questionnaire: Results of the 2019 Survey of Engineered Nanomaterial Occupational Health and Safety Practices; Table S1: Types of ENMs in Use; Table S2: Health and Safety Practices Used; Table S3: Use of Engineering Controls to Reduce Exposure to Chemical/Material Hazards; Table S4: Use of Personal Protective Equipment (PPE); Table S5: Sources Used for Information About Safe Handling of ENMs; Table S6: Resources Used to Acquire Information About Safe ENM Handling by Company Size.
