# Supplementary material for: Results of the 2019 Survey of Engineered Nanomaterial Occupational Health and Safety Practices
Source: Int J Environ Res Public Health. 2022 Jun 23;19(13):7676. doi: 10.3390/ijerph19137676 (PMC9265280; doi:10.3390/ijerph19137676)
Supplement: Supplementary file 1 [file ijerph-19-07676-s001.zip › ijerph-1761715-supplementary.pdf]

Supplementary Materials for

Results of the 2019 Survey of Engineered Nanomaterial Occupational Health and Safety Practices

Questionnaire

1. Does your company/organization develop, manufacture, use, handle, distribute, analyze, or provide services related to engineered nanomaterials?
2. Please indicate the commercial sector(s) where the engineered nanomaterials or the engineered nanomaterials-enabled products or services that your company/organization provides are intended to be used.
3. Your organization or company/organization may have multiple locations (i.e., worksite, building, facility, plant, etc.). Please respond only about the location or locations for which you have knowledge of safety and health programs. Throughout the survey we refer to these sites as "your location(s)." What is the total number of individuals (employees and contractors) who work at your location(s)?
4. How many individuals have any contact with engineered nanomaterials at your location(s)? Include both employees and contractors with either regular or occasional contact with engineered nanomaterials. *Regular Contact – Include employees that regularly handle or use engineered nanomaterials as a matter of routine during the course of their average work day. Occasional Contact – Include employees that may have infrequent, short contact with engineered nanomaterials over the course of an average work day (i.e., an employee that moves through a space where engineered nanomaterials are being handled or used such as maintenance or janitorial staff).*
5. Which of the following describes how your company/organization handles or relates to engineered nanomaterials at this location(s)?
6. In what physical forms are engineered nanomaterials handled at your location(s)?
    a. Check all that apply.)
    b. ❑ Solid, freely mobile (e.g., dry powder)
    c. ❑ Contained in an aerosol
    d. ❑ Suspended in a liquid (e.g., water, solvent)
    e. ❑ Suspended in a matrix (e.g., polymer, paste)
    f. ❑ Solid, embedded, bound, or fixed in a material or product
    g. ❑ Other, please specify:
7. On a typical day, what is the approximate quantity of engineered nanomaterials handled at your location(s)?
8. What are the different types of engineered nanomaterials at your location(s)?
9. Some government, non-profit, or for-profit organizations offer site visits or site consultations consisting of teams of experts that evaluate and provide recommendations regarding occupational safety and health practices. Since 2005 have any of your location(s) hosted such a site visit or sit consultation?
10. Who conducted the site visit(s) or site consultation(s) at your location(s)?
11. Did the site visit(s) or site consultation(s) include feedback or recommendations for handling engineered nanomaterials?

12. Was any of the feedback or recommendations for handling of engineered nanomaterials from the site visit(s) or site consultation(s) implemented?
13. Which of the following resources have you used to acquire information about how to safely handle engineered nanomaterials?
14. How has the information you acquired from any source about engineered nanomaterials been used?
15. Does your location(s) implement a safety and health program for your employees?
16. Which of the following safety and health practices are used at your location(s)? For each practice that you identify, is there separate or specific guidance for its application to engineered nanomaterials?
17. In cases where you do not have specific safety and health practices or guidelines for engineered nanomaterials, what surrogate hazards or substances do you use to inform your safety and health practices or guidelines?
18. Which of the following engineering controls are used to reduce or prevent worker exposure to any potential chemical or material hazards at your location(s)? For each engineering control you identify, is that control required when working with engineered nanomaterials?
19. Which of the following personal protective equipment (PPE) is used by individuals working with engineered nanomaterials at your location(s)?
20. Does your workforce receive **any informal or formal** training on the safe use or handling of engineered nanomaterials?
21. How is this training provided?
22. For this question, employees are categorized by their level of possible contact with engineered nanomaterials.
    Regular Contact – *Employees regularly handle or use engineered nanomaterials as a matter of routine during the course of their average work day.*
    Occasional Contact – *Employees may have infrequent, short contact with engineered nanomaterials over the course of an average work day (i.e., an employee that moves through a space where engineered nanomaterials are being handled or used such as maintenance or janitorial staff).*
    No Contact – *Employees do not have any type of contact with engineered nanomaterials.*

For each level of employee at your location(s), indicate if they receive training on the following practices regarding the safe use or handling of engineered nanomaterials. Include full-time staff, part-time staff, contractors working onsite, and/or temporary staff.

23. Is worker awareness or knowledge of engineered nanomaterials assessed at your location(s)?
24. How is it assessed?
25. What type of process emission or exposure monitoring is conducted at your location(s)?
26. When is process emission or exposure monitoring conducted?
27. Does your [company/organization] produce documents related to engineered nanomaterials?
28. What types of documents related to engineered nanomaterials are produced?
29. Does your [company/organization] have an occupational safety and health office, department, or individual?
30. How many occupational safety and health (OS&H) professionals are employed at your location(s)?
31. Altogether, how long have you worked with engineered nanomaterials?
32. What is your current position at you [company/organization]?
33. Do you have any certification in occupational safety and health (OS&H)? For example, Certified Industrial Hygienist (CIH), Certified Safety Professional (CSP), or Chemical Hazardous Material Manager (CHHM).

34. Are your responsibilities regarding occupational safety and health your:
Full-time responsibility; part-time responsibility; other duty as assigned (i.e., responsibilities are done with other responsibilities); none of the above; other.
35. Is there any other information on occupational safety and health regarding engineered nanomaterials you wish to add?

Supplemental Table S1: Types of ENMs in Use

ENM Type	n	%
Nanoparticles	29	65.9
Silver	11	25
Gold	20	45.5
Other metallic nanoparticles[1]	8	18.2
Titanium dioxide	13	29.5
Zinc oxide	14	31.8
Other metal oxide nanoparticles[2]	14	31.8
Ceramic	2	4.5
Quantum dots	12	27.3
Nanotubes, nanofibers, nanorods, or nanowires	23	52.3
Single-walled carbon nanotubes	14	31.8
Multi-walled carbon nanotubes	15	34.1
Carbon nanofibers	6	13.6
Boron nitride nanofibers	2	4.5
Semiconductor nanotubes or nanofibers	8	18.2
Semiconductor nanorods or nanofibers	6	13.6
Other metallic nanorods or nanowires[3]	1	2.3
Polymers	20	45.5
Nanosheets	16	36.4
Graphene	14	31.8
Boron nitride nanosheets	7	15.9
Other nanosheets[4]	5	11.4
Nanocrystalline cellulose	6	13.6
Nanofibrils cellulose	4	9.1
Dendrimer	4	9.1
Nanoclays	2	4.6
Other[5]	4	9.1
Missing	1	--

Note: multiple responses allowed.

[1]Carbon, nickel, copper, aluminum, zinc, titanium, iron, magnetic nanoparticles, platinum, palladium

[2]Iron metal oxides, cobalt metal oxides, cerium metal oxides, aluminum metal oxides, vanadium metal oxides, nickel metal oxides, zirconium metal oxides, silicon dioxide metal oxides, magnetic metal oxides

[3]Selenium and silver nanowires

[4]Thin films, carbon nanosheets, silicon nanosheets, transition metal dichalcogenide films

[5]Nanolipids/liposomes, DNA/RNA nanostructures

Supplemental Table S2. Health and Safety Practices Used

**General Health and Safety Practices Used**	**n**	**%**
Use of exposure controls (elimination, substitution, engineering, administrative, PPE)	44	100
Waste management/disposal procedures	41	93.2
Identification of processes or job tasks where workers may be exposed	40	90.9
Method for reporting hazards, illnesses, injuries	40	90.0
Spill cleanup procedures	39	88.6
Determination of routes of exposure	38	86.4
Evaluation of new processes/procedures for hazards	37	84.1
Assessment of need for PPE	39	88.6
Maintenance of engineering controls	37	84.1
Systematic review and update of safe use procedures	37	84.1
Review of purchase orders for possible hazardous materials	31	70.5
Assessment of effectiveness of exposure controls	28	63.6
Development of internal company/organization exposure guidelines	23	52.3
Exposure monitoring	14	31.8
Medical screening and surveillance	13	29.6
Other (radiation safety standards; site safety training guidelines)	3	6.8
Missing	1	-

Note: multiple responses allowed

Supplemental Table S3. Use of Engineering Controls to Reduce Exposure to Chemical/Material Hazards

Engineering Control	n	%	Missing (n)
Separate HVAC system	21	51.2	4
Pressure differentials	24	58.5	4
Designed or separate work areas	29	70.7	5
Cleanroom	14	35.0	5
Laboratory fume hood	19	48.7	6
Laminar low-flow ventilated enclosure	40	88.9	2
Biosafety cabinet	18	47.5	7
Glove box	15	37.5	5
Local exhaust ventilation	23	57.5	5
High-efficiency particulate air (HEPA) filtration	26	65.0	5
Ultra-low particulate air (ULPA) filtration	8	20.5	6
Working with nanomaterial in slurry or suspension	26	61.9	3

Supplemental Table S4. Use of Personal Protective Equipment (PPE)

Personal Protective Equipment	n	%
Gloves	44	100
Eye/face protection	43	97.7
Coveralls or lab coats	40	90.9
Coveralls or lab coats – woven	30	68.2
Coveralls or lab coats – non-woven	15	34.1
Respirators	16	36.4
Disposable filtering facepiece respirators	6	13.6
Elastomeric half-facepiece respirators	6	13.6
Elastomeric full-facepiece respirators	3	6.8
Powered loose-fitting facepiece respirators	1	2.3
Powered tight-fitting facepiece respirators	1	2.3
Other respirators	2	4.5
Shoe covers	13	29.6
Hair bonnets	12	27.3
No PPE required	0	0
Missing	1	-

Note: multiple responses allowed

Supplemental Table S5. Sources Used for Information About Safe Handling of ENMs

Resource	n	%
Product manufacturer information	32	72.7
Informal discussions with professional contacts or peers	25	56.8
Government publications/materials	20	45.5
NIOSH "Approaches to Safe Nanotechnology"	16	36.4
NIOSH "General Safe Practices for Working with Engineered Nanomaterials in Research Laboratories"	15	34.1
NIOSH "Current Strategies for Engineering Controls in Nanomaterial Production and Downstream Handling Processes"	10	22.7
OSHA Fact Sheet "Working Safely with Nanomaterials"	10	22.7
NIOSH "Occupational Exposure to Carbon Nanotubes and Nanofibers"	9	20.5
NIOSH "Occupational Exposure to Titanium Dioxide"	8	18.2
EPA "Control of Nanoscale Materials under the Toxic Substances Control Act"	7	15.9
EPA disposal regulations	5	11.4
Professional development course or webinar taught by NIOSH instructors	3	6.8
NIST "New NIST Reference Material Provides a Silver Lining for Nano EHS Research," 2015	3	6.8
Other Federal, state, or local government publications	3	6.8
Websites, blogs, and Internet search engines	19	43.2
Materials or publications developed by your company	18	40.9
Scientific articles, professional, or industry publications	15	34.1
Industry, scientific, or professional meetings, conferences, or tradeshows	13	29.6
International publications (e.g., Safe Work Australia)	2	4.6
Other	2	4.6
Missing	1	--

Note: multiple responses were allowed.

Supplemental Table S6. Resources Used to Acquire Information About Safe ENM Handling by Company Size

ENM Safety Resources Used	1-10 employees		11-50 employees		51-250 employees		250+ employees	
	n	%	n	%	n	%	n	%
Product manufacturer information	8	61.5	9	75.0	10	90.9	5	62.5
Informal discussions with professional contacts or peers	7	53.8	6	50.0	9	81.8	3	37.5
Government publications/material	6	46.2	4	33.3	4	36.4	6	75.0
NIOSH "Approaches to Safe Nanotechnology"	4	30.8	2	16.7	4	36.4	6	75.0
NIOSH "General Safe Practices for Working with Engineered Nanomaterials in Research Laboratories"	3	23.1	2	16.7	4	36.4	6	75.0
NIOSH "Current Strategies for Engineering Controls in Nanomaterial Production and Downstream Handling Processes"	2	15.4	3	25.0	3	27.3	2	25.0
OSHA Fact Sheet "Working Safely with Nanomaterials"	2	15.4	2	16.7	2	18.2	4	50.0
NIOSH "Occupational Exposure to Carbon Nanotubes and Nanofibers"	1	7.7	1	8.3	3	27.3	4	50.0
NIOSH "Occupational Exposure to Titanium Dioxide"	0	0.0	1	8.3	2	18.2	5	62.5
EPA "Control of Nanoscale Materials Under the Toxic Substances Control Act"	2	15.4	1	8.3	2	18.2	2	25.0
EPA disposal regulations	1	7.7	0	0.0	2	18.2	2	25.0
A professional development course or webinar taught by NIOSH instructors	0	0.0	0	0.0	1	9.1	2	25.0
NIST "New NIST Reference Material Provides a Silver Lining for Nano EHS Research"	1	7.7	0	0.0	1	9.1	1	12.5
Other Federal, state, or local government publications	1	7.7	0	0.0	1	9.1	1	12.5
Websites, blogs, and Internet search engines	8	61.5	2	16.7	6	54.5	3	37.5
Materials or publications developed by your company	4	30.8	5	41.7	6	54.5	3	37.5
Scientific articles, professional or industry publications	3	23.1	4	33.3	7	63.6	1	12.5
Industry, scientific, or professional meetings, conferences, or tradeshows	2	15.4	3	25.0	6	54.5	2	25.0

International publications (e.g., Safe Work Australia)	0	0.0	0	0.0	1	9.1	1	12.5
Missing	0	--	1	--	0	--	0	--

Note: multiple responses allowed. Percentages in each column are based on the number of respondents in each category: 1-10 employees = 13 respondents; 11-50 employees = 12 respondents; 51-250 employees = 11 respondents; 250+ employees = 8 respondents.
